# Overexpression of *AtWRKY30* Transcription Factor Enhances Heat and Drought Stress Tolerance in Wheat (*Triticum aestivum* L.)

**DOI:** 10.3390/genes10020163

**Published:** 2019-02-20

**Authors:** Mohamed A. El-Esawi, Abdullah A. Al-Ghamdi, Hayssam M. Ali, Margaret Ahmad

**Affiliations:** 1 Botany Department, Faculty of Science, Tanta University, Tanta 31527, Egypt; 2 Botany and Microbiology Department, College of Science, King Saud University, P.O. Box 2455, Riyadh 11451, Saudi Arabia; abdaalghamdi@ksu.edu.sa (A.A.A.-G.); hayhassan@ksu.edu.sa (H.M.A.); 3 Timber Trees Research Department, Sabahia Horticulture Research Station, Horticulture Research Institute, Agriculture Research Center, Alexandria 21526, Egypt; 4 UMR CNRS 8256 (B2A), Université Paris VI, 75005 Paris, France; margaret.ahmad@upmc.fr; 5 Department of Biology, Xavier University, Cincinnati, OH 45207, USA

**Keywords:** wheat, *AtWRKY30*, heat, drought, antioxidant machinery, gene expression

## Abstract

Drought and heat factors have negative impacts on wheat yield and growth worldwide. Improving wheat tolerance to heat and drought stress is of the utmost importance to maintain crop yield. WRKY transcription factors help improve plant resistance to environmental factors. In this investigation, *Arabidopsis WRKY30* (*AtWRKY30*) transcription factor was cloned and expressed in wheat. Plants growth, biomass, gas-exchange attributes, chlorophyll content, relative water content, prolines content, soluble proteins content, soluble sugars content, and antioxidant enzymes activities (catalase (CAT), superoxide dismutase (SOD), peroxidase (POX), and ascorbate peroxidase (APX)) of the *AtWRKY30*-overexpressing wheat plants were higher than those of the wild type. However, levels of electrolyte leakage, malondialdehyde, and hydrogen peroxide of the *AtWRKY30*-overexpressing wheat plants were significantly less than those of the wild-type. Additionally, the expression level of antioxidant enzyme-encoding genes and stress-responsive genes (*ERF5a*, *DREB1*, *DREB3*, *WRKY19*, *TIP2*, and *AQP7*) were significantly induced in the transgenic wheat plants in comparison with the wild type. In conclusion, the results demonstrated that *AtWRKY30* overexpression promotes heat and drought tolerance in wheat by inducing gas-exchange attributes, antioxidant machinery, osmolytes biosynthesis, and stress-related gene expression. *AtWRKY30* could serve as a potential candidate gene for improving stress tolerance in wheat.

## 1. Introduction

Abiotic stress factors such as heat and drought restrict crop productivity worldwide [1]. Abiotic stresses affect various physiological processes, leading to the over-production of toxic reactive oxygen species (ROS) in plant cells, thereby inducing oxidative damage [2,3,4,5,6]. Plants adapt through morphological, physiological, and genetic responses to overcome these harmful effects [7,8,9,10]. These adaptations include modulating antioxidant systems, root system architecture, and compatible solutes. Plant growth regulators and biostimulants have been applied to mitigate the adverse effects of abiotic stresses in different plant species [11,12,13,14,15,16,17,18,19,20,21,22]. Additionally, the transgenic approach via the overexpression of certain genes and transcription factors has proven useful in enhancing the tolerance of crops to adverse environmental stresses by the induction of downstream stress-related genes [23,24]. For instance, WRKY transcription factor family is significantly induced under stress conditions [25]. The WRKY factors also regulate the downstream-related genes and have a key role in various processes in plant species, including coping with environmental stresses [26,27,28], leaf senescence [29], and seed development [30]. Several studies have reported that WRKY-encoding genes could be stimulated by low temperatures, salt, drought, ethylene, salicylic acid, methyl jasmonate, abscisic acid, and hydrogen peroxide [31,32,33]. Additionally, overexpressing WRKY factors promoted plant tolerance to various environmental factors. For instance, *OsWRKY11* overexpression promoted drought and heat resistance in rice [34]. The overexpression of cotton genes such as *GhWRKY34*, *GhWRKY17,* and *GhWRKY41* also augmented salt and drought resistance in *Nicotiana benthamiana* [32,35,36]. Furthermore, the overexpression of wheat genes such as *TaWRKY93* and *TaWRKY19* augmented salt and drought resistance in *Arabidopsis* [37,38]. *DgWRKY4* overexpression improved salinity tolerance in chrysanthemum [39]. These transcription factors augmented plant tolerance to abiotic stresses by scavenging ROS, accumulating osmolytes, activating stress-responsive genes, and maintaining osmotic adjustment, membrane stability, and ion homeostasis [39]. However, the functions of many WRKY factors have yet to be validated in the majority of non-model plant species, particularly wheat. For instance, *AtWRKY30* gene expression conferred enhanced oxidative and salt stress tolerance in the model plant species *Arabidopsis* [40], but its function has not yet been validated or confirmed in non-model species.

Wheat (*Triticum aestivum* L.) represents one of the most essential food crops worldwide. Wheat productivity is influenced by drought and heat stresses in several regions. Various reports have revealed the important uses of the transgenic approach in augmenting drought and heat tolerance in wheat. Gao et al. [41] revealed that *TaWRKY2* overexpression augmented drought tolerance in wheat. Yu et al. [42] also demonstrated enhanced drought resistance levels in wheat lines overexpressing the bacterial *SeCspA* gene. *AtHDG11* overexpression improved drought tolerance in wheat [43]. Furthermore, the overexpression of *TaFER-5B* enhanced heat tolerance in wheat [44]. Zang et al. [45] also demonstrated that *TaPEPKR2* overexpression improved heat tolerance in wheat lines. However, further enhancement of heat and drought tolerance of wheat crops, through developing and breeding new stress-tolerant varieties, is essential to meet the food needs of the increasing population worldwide. Therefore, the current study aimed to investigate the functional role of *AtWRKY30* in promoting heat and drought tolerance in wheat as a non-model important crop. *AtWRKY30* was overexpressed in wheat, and various physiological, biochemical, and gene expression analyses were conducted for wild type and transgenic wheat in order to assess the performance of transgenic wheat lines under heat and drought conditions.

## 2. Materials and Methods

### 2.1. Plant Materials and Growth Conditions

Wheat (*Triticum aestivum* L.) Sakha-61 genotype received from the Agricultural Research Center in Egypt and the wild-type *Arabidopsis thaliana* (Col-0) received from the University of Paris VI in France were used in this study. Wheat and *Arabidopsis* seeds were surface-sterilized and left to grow at 24 °C for 5 days. Germinated seedlings were then transferred into mixed soil comprising equal volumes of peat, sand, and perlite. The seedlings were left to grow with regular irrigation under a regime of 25/19 °C, 16/8 h, and a humidity of 65%.

### 2.2. Plasmid Construction and Wheat Transformation

Total RNA was extracted from *Arabidopsis* flower using RNeasy Plant Mini kit (Qiagen, Hilden, Germany). Full cDNA was synthesized by a Qiagen Reverse Transcription kit. *AtWRKY30* coding region was then amplified and cloned, as previously reported by Scarpeci at al. [40]. In brief, *AtWRKY30* cDNA was inserted into pBinAR vector and the generated constructs (pBinAR::*AtWRKY30*) were transferred into *Agrobacterium tumefaciens* (EHA105). The resulting recombinant strain harboring the constructs was utilized to transform wheat Sakha-61 plants according to the *Agrobacterium*-mediated transformation method [46].

### 2.3. Validation Analysis of Transgenic Wheat Plants

T_0_ and T_2_ homozygous transgenic wheat genotypes were verified by quantifying the expression of *AtWRKY30* in the positive transgenics using quantitative real-time PCR (qRT-PCR). The isolation of RNA and synthesis of cDNA were conducted from the wild-type and T_0_ and T_2_ transgenic wheat genotypes, as described before. PCR reactions were carried out in triplicate by following the QuantiTect SYBR Green PCR kit manufacturer’s protocol. PCR conditions previously reported by Scarpeci et al. [40] were used. Specific primer pairs previously designed for *AtWRKY30* and the reference housekeeping gene *PP2A* [40] were used in amplification. *AtWRKY30* expression was calculated based on the 2^−ΔΔCt^ method.

### 2.4. Heat and Drought Stress Treatment

Seedlings of the wild-type and two T_2_ homozygous *AtWRKY30*-overexpressing wheat genotypes (OE-4, OE-6) were transferred into pots comprising the aforementioned mixed soil and were left to grow for 2 weeks with daily water irrigation under the same conditions as those described above. The wheat plants were then divided into three groups and were exposed to the following treatments: (1) control plants, 25/19 °C (day/light) with daily irrigation; (2) heat-stressed plants, 40/33 °C (day/night) with daily irrigation; and (3) drought-stressed plants, 25/19 °C (day/light) without watering. All treatments remained for 12 days, and plants were then collected for further analysis. The experiments were conducted in four replicates.

### 2.5. Determination of Plant Growth and Biomass, Relative Water Content, and Gas-Exchange Attributes

The shoot and root length of the collected wheat plants were recorded using a measuring scale. Shoot and root fresh weights were also calculated. Leaf relative water content (RWC) was measured as mentioned by Yamasaki and Dillenburg [47]. Stomatal conductance (*g_s_*), leaf net photosynthesis rate (*P_n_*), and transpiration rate (*E*) were determined by a gas exchange system (ADC BioScientific, Hoddesdon, UK) at 10:30 a.m., as explained by Holá et al. [48].

### 2.6. Measurement of Chlorophyll, Proline, Soluble Protein, and Soluble Sugars Contents

Leaf total chlorophyll content was calculated, as previously reported by Arnon [49]. Briefly, fresh leafy tissues (0.1 g) were homogenized in dimethyl sulfoxide and maintained in darkness for 2 days. The absorbance of homogenate was spectrophotometrically recorded at 645 and 663 nm. Leafy proline content was estimated, as previously stated by Bates et al. [50], and absorbance was read at 520 nm. To calculate leaf soluble proteins content and sugars content, a leaf sample was extracted in 100 mM Tris buffer and then centrifuged at the highest speed for 10 min. The soluble proteins content was determined using the method reported by Bradford [51]. The soluble sugar content was calculated as mentioned by Dey [52].

### 2.7. Measurement of Contents of Hydrogen Peroxide, Electrolyte Leakage, and Malondialdehyde

Hydrogen peroxide (H_2_O_2_) content was calculated by macerating 60 mg of leaf tissues in 0.1% TCA (0.6 mL). Homogenates were centrifuged at high speed and H_2_O_2_ content was calculated as previously mentioned by Velikova et al. [53]. Electrolyte leakage (EL) was estimated as previously reported by Dionisio-Sese and Tobita [54]. Malondialdehyde (MDA) content was calculated as mentioned by Rao and Sresty [55].

### 2.8. Determination of Antioxidant Enzyme Activities

Leaf tissues (0.6 g) were extracted in phosphate buffer (0.1 M, pH 7.6) and EDTA (0.5 mM). The extracts were filtered and then centrifuged at 10,000× *g* for 18 min at 5 °C. The supernatant was then utilized to estimate antioxidant enzymes activities. The activity of catalase (CAT) was measured according to Aebi [56], and absorbance was taken at 240 nm. Activities of peroxidase (POX) and superoxide dismutase (SOD) were investigated according to the method reported by Zhang [57]. Ascorbate peroxidase (APX) activity was determined as reported by Yoshimura et al. [58]. Enzyme activity was expressed as unit per milligram protein (EU mg^−1^ protein).

### 2.9. Transcriptional Analysis of Antioxidant and Stress-Responsive Genes

Quantitative real-time PCR analysis was assayed to estimate the expressions of four antioxidant genes (*CAT*, *POX*, *Mn-SOD*, and *APX*) and six stress-related genes (*ERF5a*, *DREB1*, *DREB3*, *WRKY19*, *TIP2*, and *AQP7*) in wild type and the T_2_ *AtWRKY30*-overexpressing wheat plants subjected to normal, heat, and drought treatments. RNA was extracted and cDNA was synthesized from leafy tissues, as reported above. qRT-PCR reactions were conducted following QuantiTect SYBR Green PCR kit manufacturer protocol. PCR reactions and amplification conditions for the four antioxidant genes and six stress-responsive genes were conducted as mentioned by Sheoran et al. [59] and Gao et al. [41], respectively. Specific primer pairs, previously designed for the antioxidant genes [59,60] and stress-related genes [41], were used in amplification. The wheat *Actin* gene served as an internal reference [41]. The relative expression level of genes was determined using 2^−ΔΔ*C*t^ method.

### 2.10. Statistical Analysis

One-way analysis of variance was conducted for the recorded data using SPSS v. 16 (IBM Cop., Armonk, NY, USA). Values denote the means ± SE (*n* = 4) and were significantly different at *p* ≤ 0.05.

## 3. Results and Discussion

### 3.1. Wheat Transformation and Molecular Analysis of Transgenic Lines

**Figure 1:**
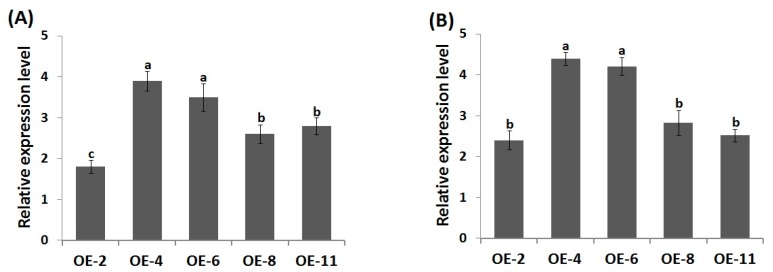
*AtWRKY30* relative expression in T_0_ (**A**) and T_2_ (**B**) overexpressed wheat lines using quantitative real-time PCR (qRT-PCR).

The genetic engineering approach has shown great potential in improving plant tolerance to environmental factors through overexpressing functional transcription factors in crop species. To investigate whether *AtWRKY30* overexpression could promote drought and heat tolerance in wheat, *AtWRKY30*-overexpressing wheat genotypes were generated by the *Agrobacterium*-mediated transformation. qRT-PCR analysis of the T_0_ and T_2_ wheat transformants revealed five lines overexpressing *AtWRKY30* (OE-2, OE-4, OE-6, OE-8, and OE-11) (Figure 1A,B). Two T_2_ lines (OE-4, OE-6) revealed the highest *AtWRKY30* expression level and were therefore used for stress analysis.

### 3.2. *AtWRKY30* Overexpression in Wheat Promotes Plant Growth and Biomass under Heat and Drought Conditions

*AtWRKY30* overexpression effect on wheat growth and biomass was investigated. Non-significant, slight differences in shoot fresh weight, shoot length, root fresh weight, and root length were observed in all wheat lines under normal growth conditions (Table 1). On the contrary, upon exposure to heat and drought stress treatments, remarkable decreases in all growth and biomass traits were detected for the wild-type and transformed wheat plants as compared with normal conditions. Wheat transgenic plants showed significantly higher increases in shoot fresh weight, shoot length, root fresh weight, and root length as compared to wild type plants (Table 1). Results reveal that *AtWRKY30* overexpression improved wheat resistance to heat and drought stress and promoted root and shoot growth.

### 3.3. *AtWRKY30* Overexpression in Wheat Enhances Gas Exchange and Leaf Relative Water Content under Heat and Drought Stress Conditions

**Figure 2:**
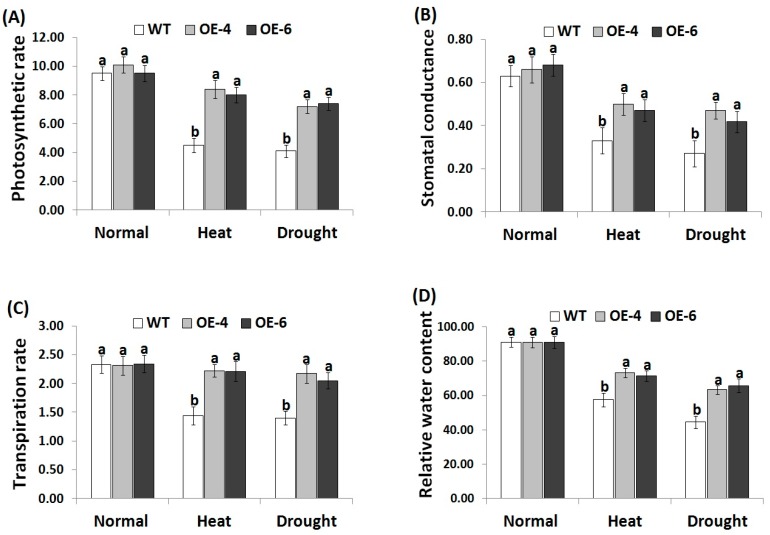
Photosynthesis rate (*P_n_*, μmol m^2^s^−1^) (**A**), stomatal conductance (*g_s_*, mol m^2^ s^−1^) (**B**), transpiration rate (*E*, mmol m^2^ s^−1^) (**C**), and relative water content (RWC, %) (**D**) of the wild type and transgenic wheat genotypes under normal, heat, and drought stress treatments. Data indicate means ± SE (*n* = 4). Different letters on the columns represent significant difference (*p* ≤ 0.05).

Gas-exchange attributes are adversely influenced by the negative impacts of environmental stresses [6]. Moreover, the leaf relative water content reveals the plants water status balance [61,62]. Decreases in the relative water content stimulate osmotic stress and limit plant growth [63]. To investigate whether the *AtWRKY30* gene transformed into wheat could enhance gas exchange characteristics and leaf relative water content in this study, the transpiration rate, photosynthetic rate, stomatal conductance, and leaf relative water content were measured in the wild type and transgenic wheat genotypes under heat and drought stress (Figure 2A–D). Under normal conditions, no significant difference was noticed in gas exchange parameters or leaf relative water content in all lines. On the contrary, under heat and stress treatments, reductions in the gas-exchange characteristics and leaf relative water content were observed for all wheat lines as compared with normal conditions. Nevertheless, *AtWRKY30-*overexpressing wheat genotypes displayed significantly higher increases in gas-exchange characteristics and leaf relative water content as compared to wild type genotype (Figure 2A–D), indicating that the transgenic wheat lines exhibited increased tolerance to heat and drought stress via promoting gas exchange parameters and leaf relative water content.

### 3.4. *AtWRKY30* Overexpression in Wheat Induces the Contents of Chlorophyll and Osmolytes under Heat and Drought Stress

**Figure 3:**
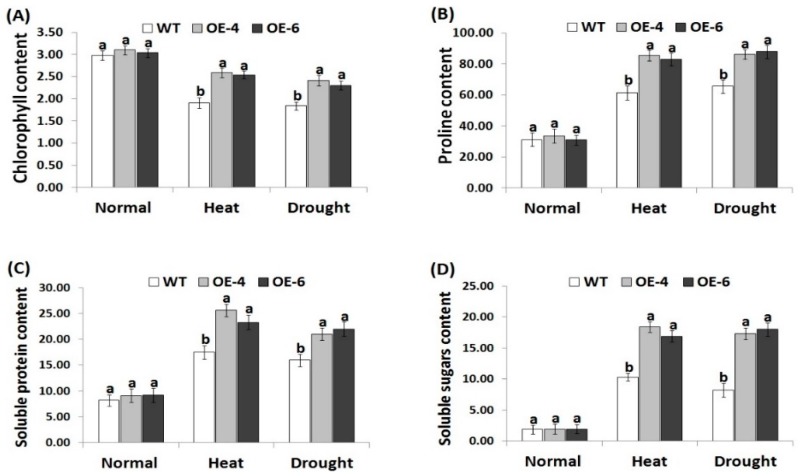
Contents of total chlorophyll (mg g^−1^ FW) (**A**), proline (µg g^-1^ FW) (**B**), soluble proteins (mg g^−1^ FW) (**C**), and soluble sugar (mg g^−1^ FW) (**D**) of wild type and transgenic wheat lines under normal, heat, and drought stress treatments. Data indicate means ± SE (*n* = 4). Different letters on the columns represent significant difference (*p* ≤ 0.05).

Chlorophyll has a key role in photosynthesis and represents a key indicator for investigating plant abiotic stress tolerance [64]. Moreover, soluble protein, soluble sugar, prolines, and other compatible solutes in plant cells maintain osmotic adjustment under stress conditions [65,66]. Proline also serves as an ROS-scavenger, while soluble proteins and sugars mitigate dehydration stress and help maintain the macromolecules structure and function [67]. Therefore, the current study investigated whether *AtWRKY30* overexpression could enhance the contents of these osmoprotectans in cells under heat and drought conditions. Under normal growth conditions, non-significantly, slight differences in chlorophyll, proline, soluble protein, and soluble sugar were recorded between the wild-type and the transformed wheat lines (Figure 3A–D). On the contrary, remarkable decrease in chlorophyll content and increases in osmolyte levels were recorded for the wild type and *AtWRKY30-*overexpressing wheat genotypes under heat and drought stress as compared with the normal growth conditions. *AtWRKY30-*overexpressing wheat genotypes had higher increases in chlorophyll and osmolytes contents in comparison with the wild type genotype under heat and drought stress treatments (Figure 3A–D), suggesting that the transformed wheat plants maintained better levels of osmolytes and chlorophyll than the wild type. Furthermore, the results reveal that *AtWRKY30* overexpression enhanced the osmoregulation ability of wheat transgenic lines to cope with dehydration stress effects. Such results are in harmony with previous findings that showed higher level of osmolytes and chlorophyll in wheat plants overexpressing stress-tolerant genes in comparison the wild type under drought stress [41,43].

### 3.5. *AtWRKY30* Overexpression in Wheat Mitigates Oxidative Stress Markers under Heat and Drought Stress Conditions

**Figure 4:**
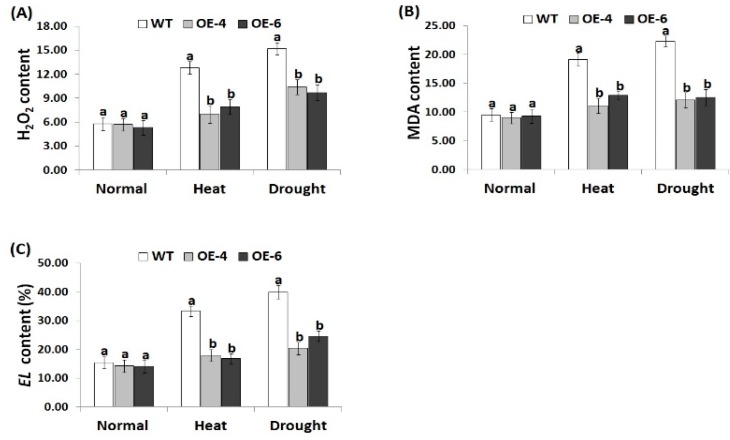
Hydrogen peroxide (H_2_O_2_, µmol g^−1^ FW) content (**A**), malondialdehyde (MDA, µmol g^−1^ FW) content (**B**), and electrolyte leakage (EL, %) (**C**) of wild type and transgenic wheat lines under normal, heat, and drought stress treatments. Data indicate means ± SE (*n* = 4). Different letters on the columns represent significant difference (*p* ≤ 0.05).

Reactive oxygen species stimulate oxidative damage in plant [6]. Electrolyte leakage reflects the cell membrane damage [68]. Additionally, MDA reflects lipid peroxidation end product damage [69]. Therefore, the current study investigated whether *AtWRKY30* overexpression in wheat could reduce ROS levels by measuring the levels of MDA, H_2_O_2_, and EL in wild type and transgenic wheat exposed to normal, heat, and drought treatments (Figure 4A–C). Under normal conditions, no obvious differences in the levels of MDA, H_2_O_2_, and EL were observed between all wheat lines. On the contrary, when exposed to heat and drought conditions, transgenic wheat showed lower levels of the oxidative stress markers in comparison with the wild type plants (Figure 4A–C). The results reveal that *AtWRKY30* overexpression in wheat reduces free radicals and the MDA level, thereby promoting wheat tolerance to heat and drought stress.

### 3.6. *AtWRKY30* Overexpression in Wheat Induces Antioxidant Enzymes Activities under Stress 

**Figure 5:**
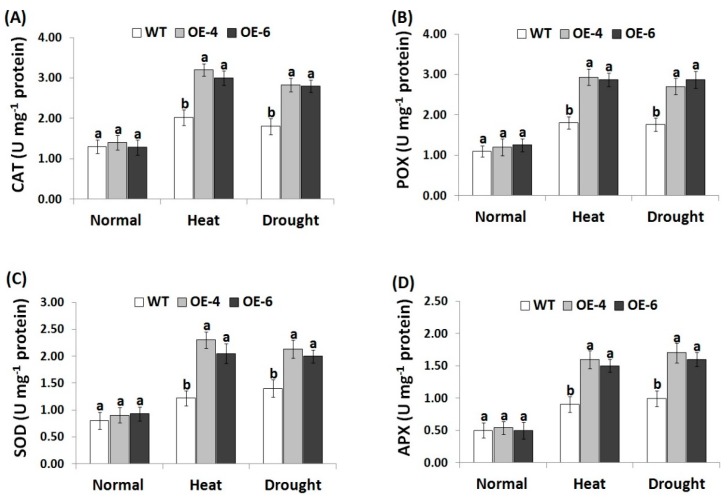
Activity of catalase (CAT) (**A**), peroxidase (POX) (**B**), superoxide dismutase (SOD) (**C**), and ascorbate peroxidase (APX) (**D**) in wild type and transgenic wheat lines under normal, heat, and drought stress treatments. Data indicate means ± SE (*n* = 4). Different letters above columns represent significant difference (*p* ≤ 0.05).

Antioxidant enzymes have important roles in scavenging ROS and promoting crop resistance to abiotic stresses [61]. In the current study, the activities of antioxidant enzymes (POX, CAT, APX, and SOD) was investigated in the wild-type and *AtWRKY30-*overexpressing wheat plants (Figure 5A–D). Non-significantly, slight differences in the four enzyme activities were recorded between wild type and transgenic wheat genotypes. On the contrary, remarkable increments in antioxidant enzyme activities were recorded for wild type and wheat transgenic genotypes under heat and drought treatments when compared to normal condition. Transgenic wheat lines showed higher antioxidant enzyme levels compared to wild type under heat and drought treatments (Figure 5A–D). The results reveal that *AtWRKY30* overexpression stimulated the antioxidant system in transgenic wheat lines to reduce the ROS-induced oxidative damage, thereby improving wheat tolerance to heat and drought conditions. Such results are in harmony with previous reports that showed higher antioxidant enzymes in wheat lines overexpressing stress-tolerant genes under heat or drought conditions [41,44].

### 3.7. *AtWRKY30* Overexpression in Wheat Induces Stress-Related Gene Expression under Stress Conditions

**Figure 6:**
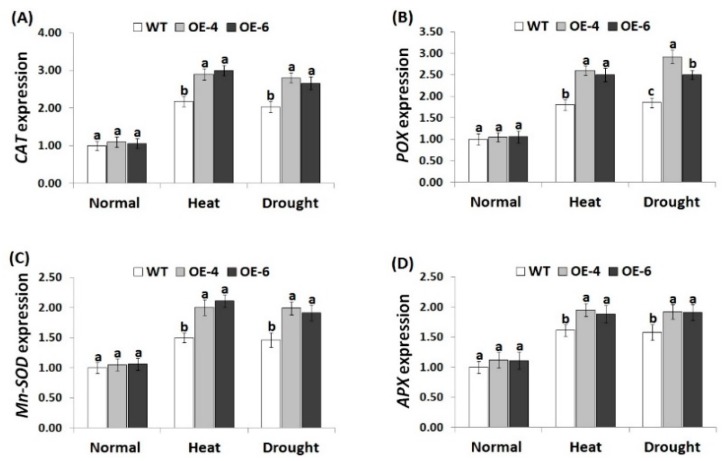
Expression levels of *CAT* (**A**), *POX* (**B**), *Mn-SOD* (**C**), and *APX* (**D**) genes in wild type and transgenic wheat lines under normal and stress treatments. Data indicate means ± SE (*n* = 4). Different letters above columns represent significant difference (*p* ≤ 0.05).

**Figure 7:**
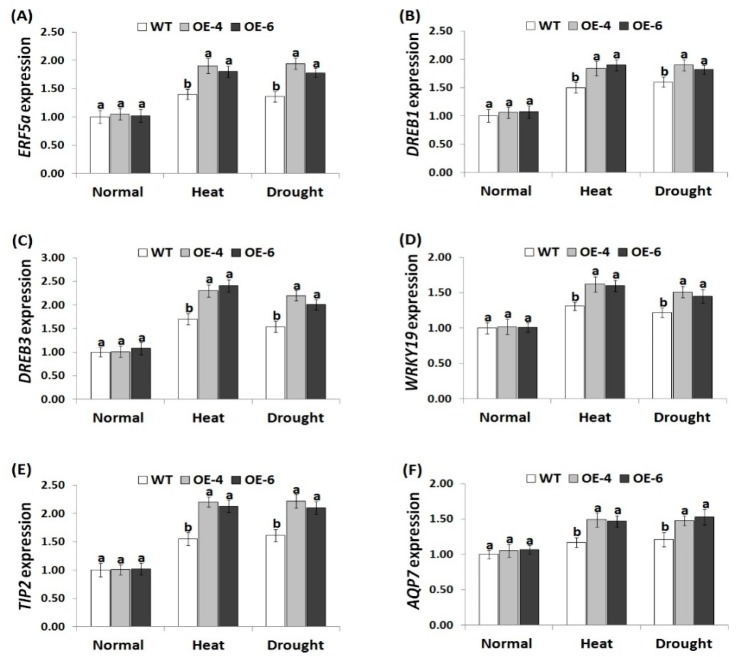
Expression levels of *ERF5a* (**A**), *DREB1* (**B**), *DREB3* (**C**), *WRKY19* (**D**), *TIP2* (**E**), and *AQP7* (**F**) genes in wild type and transgenic plants under normal and stress treatments. Data indicate means ± SE (*n* = 4). Different letters on columns denote significant difference (*p* ≤ 0.05).

To reveal the signaling regulatory roles of *AtWRKY30* in heat and drought tolerance mechanisms, expression levels of antioxidant enzymes genes (*CAT*, *POX*, *Mn-SOD*, and *APX*) and stress-related genes (*ERF5a*, *DREB1*, *DREB3*, *WRKY19*, *TIP2*, and *AQP7*) were evaluated in the wild-type and the *AtWRKY30-*overexpressing wheat plants grown under heat and drought treatments using quantitative RT-PCR. Under normal conditions, the wild type and transgenic wheat plants revealed relatively similar expression profiles for antioxidant genes (Figure 6A–D) and stress-responsive genes (Figure 7A–F). On the contrary, under heat and drought conditions, remarkable increments in the transcriptional level of the antioxidants and stress-tolerant genes were detected for the wild type and transgenic wheat genotypes as compared to normal conditions. Nevertheless, *AtWRKY30-*overexpressing wheat plants showed higher transcriptional level of the antioxidants and stress-responsive genes in comparison with wild type (Figure 6A–D and Figure 7A–F). These results reveal that *AtWRKY30* may promote heat and drought stress resistance via inducing the expression of the downstream stress-related genes involved in ROS scavenging and defense mechanisms. The results of the antioxidant genes assayed are in harmony with those of the antioxidant enzymes activity. The results are also in concordance with the previous reports that showed higher expression level of stress-responsive genes (*ERF5a*, *DREB1*, *DREB3*, *WRKY19*, *TIP2*, and *AQP7*) in *TaWRKY2*-overexpressing wheat genotypes in comparison with wild type under drought condition [41].

## 4. Conclusions

Wheat productivity is influenced by heat and drought stress worldwide. To promote wheat tolerance to heat and drought stress, the *Arabidopsis AtWRKY30* transcription factor was cloned and overexpressed in wheat. Two T_2_ transgenic wheat lines were identified and used to study heat and drought stress tolerance. The findings demonstrate that *AtWRKY30* overexpression could enhance wheat tolerance to heat and drought stress via inducing plant growth, osmolytes biosynthesis, gas-exchange parameters, antioxidant enzymes activity, ROS scavenging, and stress-related gene expression. *AtWRKY30* would be a potential candidate for augmenting wheat tolerance to heat and drought stresses.

## Figures and Tables

**Table 1:** Growth and biomass of the wild type and *AtWRKY30*-overexpressing wheat plants grown under normal, heat, and drought stress conditions.

Treatments	Lines	SL (cm/plant)	RL (cm/plant)	SFW (g/plant)	RFW (g/plant)
Normal	WT	31.11 ± 1.13a	23.22 ± 1.32a	2.81 ± 0.11a	2.13 ± 0.17a
	OE-4	32.21 ± 1.67a	24.81 ± 1.18a	2.98 ± 0.25a	2.41 ± 0.21a
	OE-6	31.19 ± 1.81a	24.44 ± 1.13a	3.02 ± 0.18a	2.32 ± 0.18a
Heat	WT	25.11 ± 1.62b	16.35 ± 1.38b	2.17 ± 0.12b	1.79 ± 0.17b
	OE-4	30.71 ± 1.42a	22.11 ± 1.59a	2.76 ± 0.16a	2.11 ± 0.18a
	OE-6	29.11 ± 1.51a	21.62 ± 1.57a	2.66 ± 0.12a	2.13 ± 0.21a
Drought	WT	21.31 ± 1.12b	12.66 ± 1.22b	2.04 ± 0.22b	1.44 ± 0.11b
	OE-4	27.13 ± 1.11a	16.99 ± 1.04a	2.19 ± 0.16a	1.83 ± 0.21a
	OE-6	26.51 ± 1.32a	17.11 ± 1.12a	2.17 ± 0.21a	1.85 ± 0.17a

SL, shoot length; RL, root length; RFW, root fresh weight; SFW, shoot fresh weight; WT, wild-type; OE, overexpressing plants. Values represent means ± SE (*n* = 4). Different letters next to the numbers under the same treatment denote significant difference between lines (*p* ≤ 0.05).
